# Advancing immunotherapy for esophageal cancer: decoding the roles of PD-L1, TME, and tumor-intrinsic biomarkers

**DOI:** 10.3389/fimmu.2025.1679365

**Published:** 2025-11-18

**Authors:** Jiakang Ma, Dangrou Wu, Yongxuan Liu, Guangping Zhang

**Affiliations:** 1 Henan Key Laboratory of Cancer Epigenetics, Cancer Hospital, The First Affiliated Hospital, and College of Clinical Medicine of Henan University of Science and Technology, Luoyang, China; 2 Department of Pathology, The First Affiliated Hospital of Henan University of Science and Technology, Luoyang, China

**Keywords:** esophageal cancer, programmed death-ligand 1, tumor immune microenvironment, predictive biomarkers, immunotherapy, chemoimmunotherapy

## Abstract

Esophageal squamous cell carcinoma (ESCC) remains a global health challenge, with immune checkpoint inhibitors (ICIs) reshaping therapeutic strategies. However, heterogeneous responses underscore the urgent need for robust predictive biomarkers. While PD-L1 expression remains the most widely used marker, its limitations, including spatial heterogeneity and inducible expression, have prompted exploration of alternative and composite indicators. Recent advances highlight the predictive potential of tumor immune microenvironment (TME) features such as tumor-infiltrating lymphocytes (TILs), tertiary lymphoid structures (TLSs), stromal maturity, and T cell–inflamed gene expression profiles. Concurrently, tumor-intrinsic biomarkers, including microsatellite instability, tumor mutational burden, neoantigen load, and chromosomal alterations—have shown promise in stratifying immunotherapy responders. Multi-omics approaches, liquid biopsies, and integration of host factors such as gut microbiota are emerging to refine patient selection. This review comprehensively examines evolving biomarkers and therapeutic trials, emphasizing the need for integrative precision strategies to optimize immunotherapy efficacy in ESCC.

## 1 Introduction

Esophageal squamous cell carcinoma (ESCC), ranking seventh in global incidence and sixth in cancer-related mortality (1), is typically diagnosed at advanced stages with limited treatment efficacy. Immune checkpoint inhibitors (ICIs) have shown promising efficacy and manageable safety in advanced ESCC, as evidenced by trials such as KEYNOTE-181 (2), ESCORT (3), and KEYNOTE-590 (4), leading to their integration from later-line to first-line and neoadjuvant settings. However, the clinical benefit of ICIs is heterogeneous and influenced by multiple factors, including tumor immunogenicity, the tumor immune microenvironment (TIME), and host-related characteristics. Not all patients derive satisfied responses, underscoring the urgent need for robust predictive biomarkers to identify likely responders (5).

Programmed death-ligand 1 (PD-L1) expression remains the most widely recognized biomarker for predicting response to ICIs. Nevertheless, its predictive accuracy is limited, and a substantial proportion of patients with high PD-L1 expression still fail to respond to treatment (5). Ongoing research efforts have identified two broad categories of emerging biomarkers with potential predictive value in immunotherapy. The first category encompasses features of the TIME, such as tumor-infiltrating lymphocytes (TILs), the presence of tertiary lymphoid structures (TLS), and T cell–inflamed gene expression profiles. These factors reflect the immune contexture within the tumor and its potential responsiveness to immune modulation (6). The second category involves molecular characteristics intrinsic to tumor cells, including high microsatellite instability (MSI-H)/deficient mismatch repair (dMMR), tumor mutational burden (TMB), and neoantigen load, all of which are associated with enhanced immunogenicity and increased likelihood of immune recognition (7). Additional exploratory biomarkers include serum non-coding RNAs, DNA methylation signatures, and components of the gut microbiome, though their predictive relevance in ESCC remains to be validated through larger, high-quality datasets (8, 9). This review aims to provide a comprehensive synthesis of the current understanding of immunotherapeutic biomarkers in ESCC, with particular emphasis on PD-L1, the TIME, tumor cell–related molecular features, and their clinical implications in guiding immunotherapy strategies.

## 2 Mechanistic and spatial heterogeneity of PD-L1 expression in ESCC

PD-L1, a pivotal immunoregulatory molecule, is broadly expressed across solid tumors and infiltrating immune cells, and remains the most extensively investigated biomarker for predicting clinical responses to ICI therapy. In the KEYNOTE-181 trial, pembrolizumab improved median overall survival (OS) to 12.5 months in ESCC patients with PD-L1 combined positive score (CPS) ≥10, compared to 10.0 months in the overall cohort (2). Consistent outcomes were observed in the KEYNOTE-590 subgroup with CPS ≥10 (4). Nevertheless, PD-L1 fails to serve as a definitive predictor of therapeutic efficacy. In the ESCORT trial, demonstrated no significant correlation between PD-L1 expression levels and clinical response parameters (3), and responses have been reported even in PD-L1 negative patients (10). Despite its clinical utility, PD-L1 as a predictive biomarker is limited by spatial heterogeneity and TME complexity (11, 12). Expression may differ across tumor regions and metastatic sites, leading to sampling bias. Moreover, PD-L1 upregulation is not exclusively IFN-γ–driven; alternative pathways, such as PTEN loss or EGFR mutations, can induce expression independent of antitumor immunity (13). Its inducible nature also results in dynamic changes under therapeutic or immune pressure, undermining its stability as a predictive marker. As immunotherapy shifts toward combination regimens, reliance on PD-L1 alone has diminished. These limitations underscore the need for integrative biomarker strategies, incorporating tumor-infiltrating lymphocytes, T cell–inflamed gene signatures, or composite immune scores, to improve stratification and optimize treatment efficacy in ESCC and beyond (14).

## 3 The tumor immune microenvironment

### 3.1 Tertiary lymphoid structures

TILs, particularly CD8^+^ cytotoxic T cells, are central mediators of anti-tumor immunity and have emerged as prognostic and predictive biomarkers across malignancies (15–17). Immunophenotyping based on CD3^+^ and CD8^+^ T cell density and localization delineates tumors into immune-desert, inflamed, immune-excluded, and immunosuppressed categories, with ICIs demonstrating greater efficacy in inflamed phenotypes (18). Integrating PD-L1 expression with TIL density improves predictive accuracy for PD-1/PD-L1 blockade, with PD-L1^+^/TIL^+^ tumors showing superior responses (19). In ESCC, however, the prognostic and predictive roles of TILs remain to be fully elucidated, necessitating refined stratification strategies based on TIL subtypes and additional immune markers. Eosinophils, beyond their role in eosinophilic esophagitis, are found to inversely correlate with lymph node metastasis in ESCC and may serve as dynamic markers of immunotherapy response (20, 21). Tumor-associated macrophages (TAMs), especially M2-like subsets, drive immune evasion by secreting IL-10 and TGF-β, fostering Treg recruitment and CTL inhibition (22), with high TAM density correlating with poor prognosis in ESCCs (23). Functional exhaustion of NK cells in ESCC is characterized by diminished granzyme B and activating receptors (NKp30, NKG2D), driven by IL-6/IL-8–mediated STAT3 activation, and correlates with disease progression (24). Myeloid-derived suppressor cells (MDSCs) further contribute to immunosuppression via ROS, arginase-1, and nitric oxide production (23, 25, 26). Collectively, these immune components establish a profoundly immunosuppressive tumor microenvironment, attenuating cytotoxic responses and limiting immunotherapeutic efficacy. TLSs are ectopic lymphoid aggregates formed in non-lymphoid tissues, including tumor sites and regions of chronic inflammation, and are composed of diverse immune cell populations (27). While studies directly examining the role of TLSs in ESCC remain limited, accumulating evidence indicates that TLSs are robust predictors of ICI efficacy in several tumor types, independent of PD-L1 expression status (28). Notably, a recent study in melanoma demonstrated that patients exhibiting high TLS-related gene signature scores (TLS-H) experienced significantly improved survival following CTLA-4 blockade, highlighting the critical contribution of TLSs to the maintenance of effective anti-tumor immune responses (29). The potential predictive value of TLSs in ESCC immunotherapy warrants further investigation.

### 3.2 Tumor stromal maturity

TSM is assessed based on the organization and morphology of collagen fibers and the presence of myxoid changes within the tumor stroma, and is generally categorized into mature, intermediate, and immature subtypes. TSM has been strongly associated with tumor metastasis and prognosis in malignancies such as colorectal cancer (30) and gastric cancer (31). Immature stromal subtype in ESCC is correlated with more aggressive biological behavior and poorer clinical outcomes. Furthermore, TSM was found to be associated with PD-L1 expression, suggesting its potential utility as a predictive biomarker for immunotherapeutic efficacy in ESCC (32). In head and neck squamous cell carcinoma, specific subtypes of cancer-associated fibroblasts (CAFs) have also been implicated in modulating immunotherapy responses, further highlighting the role of stromal components as determinants of treatment efficacy (33). Importantly, recognizing TSM may help guide therapeutic stratification, wherein patients with immature stroma—characterized by a dense, disorganized matrix and immunosuppressive fibroblast phenotypes—might benefit from combined stromal-targeting and immunotherapeutic approaches (32, 34). Thus, integrating TSM assessment into routine pathological evaluation could enhance precision in tailoring immunotherapy regimens.

### 3.3 T cell–inflamed gene expression profile

The T cell–inflamed gene expression profile (GEP) captures the immunogenic characteristics of the TME (35). An RNA-based transcriptomic analysis of baseline tumor samples from patients treated with pembrolizumab revealed that the T cell–inflamed GEP comprises interferon-γ-responsive genes associated with antigen presentation, chemokine expression, cytotoxic activity, and adaptive immune resistance (36). Cristescu et al. performed whole-genome and RNA expression profiling of patients with advanced solid tumors and melanoma across four KEYNOTE clinical trials. Based on combined stratification of tumor mutational burden and GEP levels, they identified four distinct clinical response groups, with the TMB^high^/GEP^high^ cohort exhibiting the strongest therapeutic responses, thus positioning T cell–inflamed GEP as a potential predictive biomarker for immunotherapy efficacy (37). Therefore, the integration of TMB and GEP holds promise as a robust strategy to guide precision immunotherapy, particularly in anti–PD-1 contexts.

## 4 Molecular characteristics of tumor cells as predictive biomarkers

### 4.1 Microsatellite instability and mismatch repair deficiency

Microsatellite instability (MSI) refers to alterations in the length of microsatellites—short tandem repeats scattered throughout the genome—resulting in the appearance of novel alleles. The DNA mismatch repair (MMR) system, composed of a set of highly conserved genes and their encoded enzymes—including MLH1, MSH2, MSH6, and PMS2—functions to correct base-pair mismatches during DNA replication. Deficiency in MMR (dMMR) impairs this repair capacity, thereby promoting the accumulation of replication errors and contributing to MSI development (38). High-level microsatellite instability (MSI-H) is associated with aberrations in cancer-related genes, facilitating tumorigenesis. Moreover, MSI-H tumors often display increased neoantigen expression and TIL density, both of which enhance responsiveness to ICIs (39). Despite its immunologic relevance, the prevalence of MSI-H in ESCC remains low, ranging from 0% to 27% across studies (40), with discrepancies likely stemming from variations in MSI-H definitions, assay loci, and detection thresholds. Based on findings from multiple clinical trials involving various solid tumors (38, 41), pembrolizumab, a PD-1 inhibitor, was approved for the treatment of MSI-H/dMMR-positive solid tumors irrespective of histology (42), encompassing MSI-H/dMMR esophageal cancer. Although only a small subset of ESCC patients may qualify under this indication, it offers a potential immunotherapy avenue for PD-L1–negative patients.

### 4.2 Tumor mutational burden and neoantigen burden

**Figure 1:**
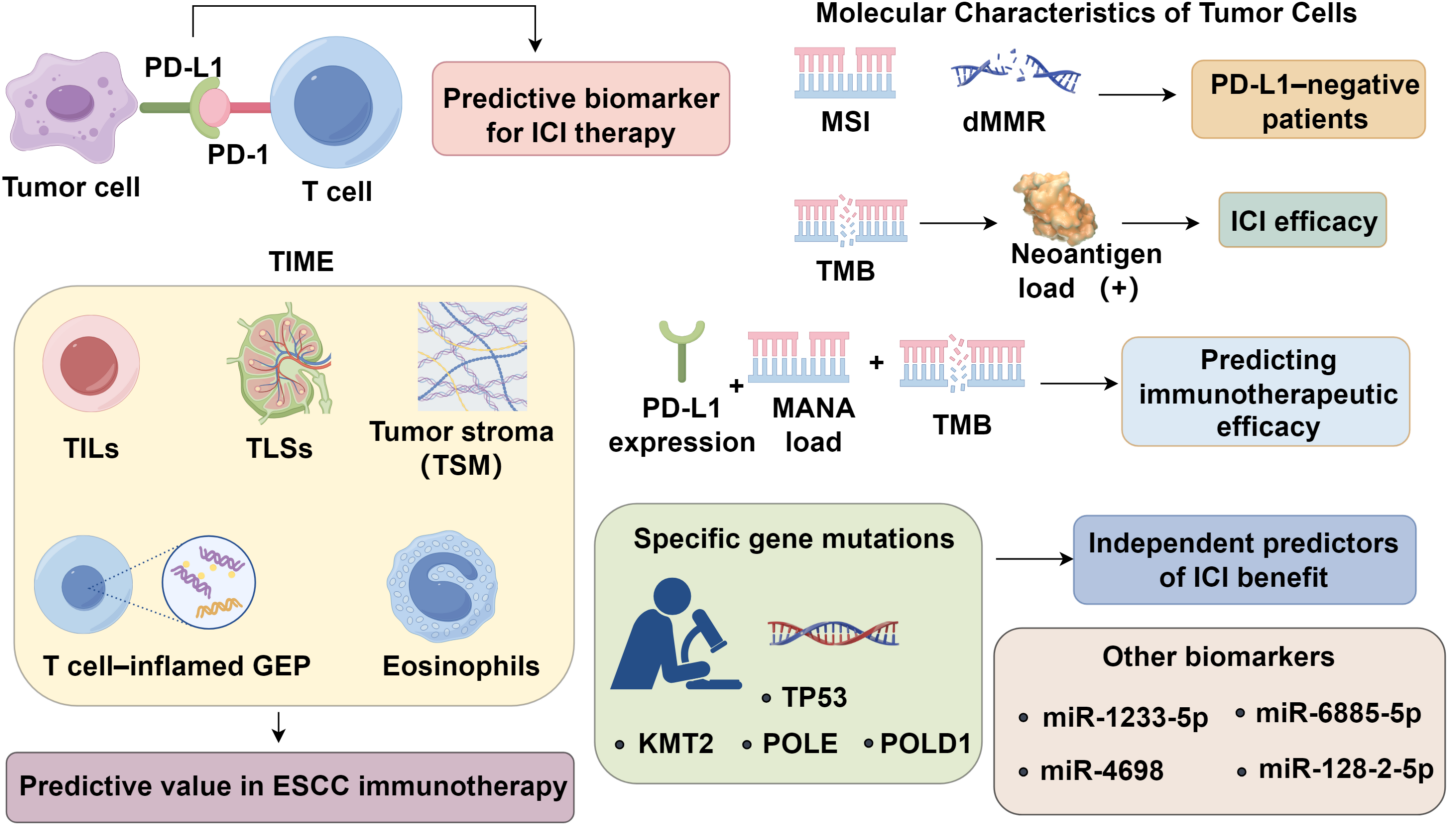
Predictive biomarkers for esophageal cancer immunotherapy.

TMB quantifies the number of somatic nonsynonymous mutations within a defined genomic region. A higher TMB correlates with increased neoantigen load, enhancing immunogenicity and the likelihood of ICI efficacy. In the multicenter Phase II KEYNOTE-158 trial involving diverse advanced solid tumors (43), a threshold of 10 mutations per megabase (Mut/Mb) was established to define TMB-high (TMB-H) status. Patients with TMB-H demonstrated superior clinical responses to pembrolizumab monotherapy, achieving an objective response rate (ORR) of 30.3%, compared to 6.7% in TMB-low (TMB-L) counterparts. These findings made pembrolizumab approved for TMB-H tumors patients with progressive, unresectable, or metastatic disease regardless of tumor origin. A pan-cancer analysis further confirmed that high TMB was associated with improved response rates and prolonged survival following ICI therapy (44). Nonetheless, TMB varies widely across cancer types, and the top 20% threshold for defining TMB-H differs significantly between malignancies (45), precluding the use of a uniform cutoff. Notably, a consensus threshold for TMB-H specific to esophageal cancer remains undefined. Somatic mutations may give rise to tumor neoantigens, which represent biomarkers of highly immunogenic tumors Neoantigen burden has been correlated with patient survival and ICI responsiveness across various cancer types (46, 47). In ESCC patients treated with the anti–PD-1 antibody camrelizumab (SHR-1210), both TMB and mutation-associated neoantigens (MANAs) were positively associated with therapeutic response (48). A composite biomarker approach combining PD-L1 expression, MANA load, and TMB may offer a promising strategy for predicting immunotherapeutic efficacy (**Figure 1**).

### 4.3 Chromosomal amplification and specific gene mutations

Chromosomal amplification events have emerged as potential predictive markers of ICI resistance. Notably, ESCC patients exhibit amplification of chromosome 11q13, which is associated with advanced tumor stage. Mechanistically, 11q13 amplification often includes genes such as CCND1, FGF3/4/19, and ORAOV1. Overexpression of CCND1 can activate the CDK4/6-Rb axis, leading to cell cycle progression and immune evasion through decreased tumor immunogenicity. Additionally, CDK4/6 activation has been shown to downregulate MHC class I expression, thereby impairing antigen presentation and cytotoxic T cell recognition. These effects collectively blunt antitumor immunity and may underlie the reduced responsiveness to ICIs observed in 11q13-amplified ESCC. Evidence from a clinical trial of toripalimab in esophageal cancer suggests that 11q13 amplification may serve as a negative predictor of response to PD-1 blockade in metastatic ESCC (49). Mutations in *TP53* are implicated in the pathogenesis of numerous cancers, including esophageal carcinoma. However, the predictive value of *TP53* mutations for immunotherapy response remains ambiguous and may be context-dependent. While *TP53* mutations have been associated with positive ICI response in breast and lung adenocarcinomas, they correlate with poor response in gastric, colorectal, and head and neck squamous cell carcinomas (50). In addition, mutations in genes such as *KMT2* (51), *POLE*, and *POLD1* (52) have been identified as independent predictors of ICI benefit across multiple tumor types, yet their mutation frequency in ESCC is exceedingly low. The relevance of other ESCC-associated gene alterations in shaping immunotherapeutic outcomes remains to be elucidated.

### 4.4 Other biomarkers

Several noncoding RNA and serum-based biomarkers have shown potential in predicting immunotherapy efficacy. For example, patients with advanced ESCC who responded to second-line nivolumab therapy exhibited reduced baseline levels of miR-1233-5p, as well as decreased expression of miR-6885-5p, miR-4698, and miR-128-2-5p during treatment, suggesting that specific circulating microRNAs may serve as predictive indicators (53). In other cancers, circulating long non-coding RNAs (54) and circular RNAs (55) have also been linked to ICI responsiveness. Elevated levels of peripheral biomarkers—such as serum albumin, neutrophils, inflammatory cytokines, and C-reactive protein—during nivolumab treatment have been associated with disease progression (56). Additionally, methylation profiling of CpG sites has been used to develop risk-scoring models with prognostic and predictive value for PD-1 inhibitor therapy (57). Emerging evidence also implicates host microbiota and their metabolic products as key modulators of ICI efficacy (58). Furthermore, host factors such as age and obesity may also influence immunotherapeutic outcomes (59). The identification and validation of novel predictive biomarkers and composite models remain active areas of investigation.

## 5 Immunotherapy

### 5.1 Pembrolizumab

PD-1, expressed on T, B, and NK cells, maintains immune tolerance by regulating T cell differentiation (60). Its ligand PD-L1 is overexpressed in various cancers and suppresses T cell activity via PD-1 binding. PD-1/PD-L1 inhibitors are the main immunotherapy in ESCC. The PALACE-1 trial evaluated neoadjuvant chemoradiotherapy (nCRT) combined with pembrolizumab in 20 patients with resectable ESCC, all of whom experienced treatment-related adverse events (AEs) (61). Eighteen underwent surgery, with a pathologic complete response (pCR) achieved in 56%. While nCRT improves pCR rates, survival benefits remain uncertain, prompting an ongoing phase II multicenter trial (NCT04807673). For advanced esophageal cancer post-first-line chemotherapy, the KEYNOTE-181 trial demonstrated that pembrolizumab significantly prolonged median OS versus chemotherapy in patients with PD-L1 CPS ≥10, with fewer AEs (62), leading to its approval by the NMPA in June 2020. Furthermore, the KEYNOTE-590 trial established the superiority of first-line chemoimmunotherapy over chemotherapy alone. Pembrolizumab plus chemotherapy was first-line treatment and reduced mortality risk by 49% and improved ORR in ESCC (4, 63) (**Table 1**).

**Table 1:** Clinical trials of ICIs in esophageal cancer.

Clinical Trial	Drug(s)	Setting	Outcome
KEYNOTE-181	Pembrolizumab	2nd-line metastatic ESCC	Prolonged OS in PD-L1 CPS ≥10 patients (9.3 vs. 6.7 months); fewer AEs
KEYNOTE-590	Pembrolizumab + chemotherapy	1st-line unresectable/metastatic ESCC	Reduced mortality risk by 49%; improved ORR (37.3% vs. 20.0%)
ESCORT	Camrelizumab	2nd-line metastatic ESCC	Improved OS (8.3 vs. 6.2 months), benefit in PD-L1 ≥1%
CheckMate-577	Nivolumab	Adjuvant post-nCRT	Prolonged DFS and metastasis-free survival, independent of PD-L1 or histology
ATTRACTION-03	Nivolumab	2nd-line metastatic ESCC	Extended median OS versus taxane chemotherapy
CheckMate-648	Nivolumab ± Ipilimumab	1st-line unresectable/metastatic ESCC	Improved OS regardless of PD-L1 status
NICE	Camrelizumab + albumin-paclitaxel + carboplatin	Neoadjuvant for resectable ESCC	98.0% R0 resection rate; 39.2% pCR; manageable toxicity
ESPRIT	Camrelizumab + paclitaxel + nedaplatin	Neoadjuvant ESCC	ORR 38.1%; 57.14% pCR in 7 surgical cases; no treatment-related mortality
ESCORT-1st	Camrelizumab + chemotherapy	1st-line unresectable/metastatic ESCC	Prolonged OS and PFS regardless of PD-L1; reduced death/progression risk by ~48–49%
JUPITER-06	Toripalimab + chemotherapy	1st-line advanced/metastatic ESCC	42% reduction in progression/death risk; improved OS and ORR
KEEP-G03	Sintilimab + triplet chemotherapy	Neoadjuvant for resectable ESCC	26.7% pCR; 53.3% mPR; 100% R0 resection rate
ORIENT-15	Sintilimab + chemotherapy	1st-line advanced ESCC	Median OS improved; 37% reduction in death risk; better patient-reported outcomes

### 5.2 Nivolumab

nCRT followed by surgery has demonstrated the most substantial survival benefit for resectable, locally advanced esophageal cancer (64, 65). However, recurrence remains problematic. The CheckMate-577 trial (66, 67) addressed this by evaluating adjuvant nivolumab in patients with residual disease post-nCRT and R0 resection, revealing prolonged disease-free survival and metastasis-free survival, independent of PD-L1 expression or histology. In advanced settings, the ATTRACTION-03 trial (68, 69) established nivolumab as a second-line standard for ESCC, extending median overall survival versus taxane chemotherapy. Furthermore, the CheckMate-648 study (70)showed that both nivolumab plus chemotherapy and nivolumab plus ipilimumab significantly improved overall survival compared to chemotherapy alone in unresectable/metastatic ESCC, with consistent benefits regardless of PD-L1 status. Collectively, these trials support nivolumab’s role across multiple disease stages and treatment lines in ESCC.

### 5.3 Camrelizumab

Camrelizumab-based regimens have demonstrated promising efficacy and safety in both neoadjuvant and palliative settings for ESCC. The NICE study (71) enrolled 60 patients with resectable, locally advanced thoracic ESCC and multi-station lymph node metastases. Neoadjuvant camrelizumab combined with albumin-bound paclitaxel and carboplatin yielded a 98.0% R0 resection rate and a 39.2% pCR, with manageable toxicity. Similarly, the ESPRIT study (72) reported an objective response rate of 38.1% and 57.14% pCR in 7 surgical cases following camrelizumab plus paclitaxel and nedaplatin, with no treatment-related mortality. In the palliative setting, the ESCORT study (3) showed improved median OS with second-line camrelizumab, particularly in PD-L1 ≥1% patients. Furthermore, the ESCORT-1st trial (73) established camrelizumab combined with chemotherapy as an effective first-line treatment in unresectable or metastatic ESCC, prolonging OS and progression-free survival (PFS) regardless of PD-L1 expression. Notably, in patients with tumor proportion scores ≥10, camrelizumab reduced death and progression risks, while even PD-L1–negative patients derived clinical benefit.

### 5.4 Toripalimab and sintilimab

Perioperative toripalimab combined with neoadjuvant chemoradiotherapy (nCRT) was evaluated in 20 patients with locally advanced ESCC (74). Among them, 13 underwent surgery, yielding a pathological complete response (pCR) rate of 54%. Lymphopenia and leukopenia were the most common adverse events (AEs), with no postoperative recurrences observed at a 6-month median follow-up. These findings support the feasibility and tolerability of toripalimab–nCRT regimens in resectable ESCC, though long-term efficacy warrants further validation. In metastatic ESCC, toripalimab monotherapy showed preliminary activity, and the phase III JUPITER-06 trial (75) demonstrated that toripalimab plus chemotherapy significantly improved median OS and ORR by 17.2%, reducing the risk of progression or death by 42% versus chemotherapy alone. Similarly, the KEEP-G03 study (76) assessed neoadjuvant sintilimab with triplet chemotherapy (liposomal paclitaxel, cisplatin, and S-1) in resectable ESCC. Among 15 surgical patients, all achieved R0 resection; pCR and major pathological response (mPR) rates were 26.7% and 53.3%, respectively. Grade 3–4 AEs included leukopenia, neutropenia, and anemia, with no grade 5 events or surgery delays. In the ORIENT-15 phase III trial (77), sintilimab plus chemotherapy significantly prolonged median OS and PFS in advanced ESCC, reducing death and progression risks by 37% and 44%, respectively, with a manageable safety profile. Patient-reported quality-of-life outcomes also favored the sintilimab combination arm, underscoring its clinical benefit.

## 6 Conclusion

The integration of immune checkpoint inhibitors has significantly expanded the therapeutic arsenal against esophageal squamous cell carcinoma, yet substantial heterogeneity in response underscores the limitations of current biomarkers—particularly PD-L1—as standalone predictive tools. The immune landscape of ESCC is shaped by dynamic and complex interactions among tumor-intrinsic genetic alterations, immune-infiltrating cell populations, stromal architecture, and systemic host factors, all of which modulate immunotherapeutic responsiveness. Accumulating evidence supports the incorporation of tumor mutational burden, microsatellite instability, neoantigen load, and gene expression signatures as adjunctive or composite biomarkers that may refine patient stratification beyond PD-L1 status. Similarly, immune-rich microenvironments defined by TILs, TLSs, and mature stroma portend more favorable outcomes and offer additional layers of predictive value.

Moving forward, precision immunotherapy in ESCC will require the standardization and clinical validation of multi-parametric biomarker platforms—spanning genomics, transcriptomics, epigenetics, proteomics, and microbiome profiling. Therapeutically, rational combinations involving ICIs and agents targeting the tumor stroma, immunosuppressive myeloid cells, or oncogenic signaling pathways hold promise for overcoming resistance. Finally, future clinical trials must prioritize biomarker-driven design and incorporate patient-reported outcomes to ensure personalized, effective, and tolerable treatment strategies. By decoding the complex interplay between tumor biology and immune contexture, the field is poised to transform ESCC management through next-generation immunotherapy.
